# Factors associated with birth preparedness and complication readiness among pregnant women attending government health facilities in the Bamenda Health District, Cameroon

**DOI:** 10.11604/pamj.2021.39.47.18736

**Published:** 2021-05-19

**Authors:** Yunga Patience Ijang, Pierre Marie Tebeu, Claude Nkfusai Ngwayu, Mbinkar Adeline Venyuy, Tchinda Basile, Fala Bede, Frankline Sevidzem Wirsiy, Samuel Nambile Cumber

**Affiliations:** 1 Department of Public Health, School of Health Sciences, Catholic University of Central Africa, Box 1110, Yaoundé, Cameroon,; 2 Department of Public Health, School of Nursing and Public Health, University of Kwa-Zulu Natal, Durban, South Africa,; 3 Cameroon Baptist Convention Health Services (CBCHS), Yaoundé, Cameroon,; 4 Médecins Sans Frontières (MSF) Operational Centre Brussels (OCB), 11 Avenue Massamba, P.O. Box: 15699-Kin 1, Kinshasa, Democratic Republic of Congo,; 5 Cameroon Society of Epidemiology (CaSE), Yaoundé, Cameroon,; 6 Institute of Health and Caring Sciences, The Sahlgrenska Academy at University of Gothenburg, Gothenburg, Sweden,; 7 Faculty of Health Sciences, University of the Free State, Bloemfontein, South Africa

**Keywords:** Factors, birth preparedness, complication readiness, Bamenda Health District, Cameroon

## Abstract

**Introduction:**

birth preparedness and complication readiness (BPCR) intervention should greatly have an impact on the reduction of maternal mortality if implemented properly at all levels. Responsibility for BPCR must be shared among all safe motherhood stakeholders-because a coordinated effort is needed to reduce the delays that contribute to maternal and newborn deaths. This study aimed to assess the factors associated with birth preparedness and complication readiness among pregnant women attending government health facilities in the Bamenda Health District.

**Methods:**

this was a cross-sectional analytic study. The study period was 30^th^ October - 30^th^ November, 2016. A total of 345 pregnant women of ≥ 32 weeks gestational age seen at the antenatal consultation (ANC) units were recruited. The dependent variable was birth preparedness and complication readiness while the independent variables were the socio-demographic and reproductive health characteristics. Frequency distributions were used to determine the awareness and practice and logistic regression at 95% confidence interval (CI) and p<0.05 to identify the factors that favour birth preparedness and complication readiness.

**Results:**

the most likely factors that favour birth preparedness and complication readiness were monthly income (Odds Ratio (OR) = 2.94, (1.39, 6.25), p = 0.005) and the number of antenatal care visits (OR = 2.16, (1.18, 3.90), p = 0.013).

**Conclusion:**

majority of the women in this study were not prepared for birth/complications. The factors most associated with birth preparedness and complication readiness were monthly income and number of antenatal care visits.

## Introduction

Maternal mortality was considered a problem by the international public health community in 1987 which led to the initiation of various programmes and strategies to reduce the growing number of maternal deaths. These strategies included the training of traditional birth attendants (TBA), and later identifying pregnant women at risk to optimise care. Without much benefits obtained, the traditional antenatal consultation has been replaced with the focused antenatal consultation (FANC) package where care becomes more individualised and goal-oriented. Birth preparedness and complication readiness are one of the goals of the FANC adopted by the World Health Organisation (WHO) in 2001. It has as objective to minimize disorganisation at the time of birth or in an emergency, thus ensuring timely and appropriate care [1]. For maternal deaths are thought to occur due to three main delays; delays in deciding to seek care, delays in reaching care, and delays in receiving care. These delays have many causes, including logistic and financial concerns, unsupportive policies, and gaps in services, as well as inadequate community and family awareness and knowledge about maternal health issues [2]. With adequate population coverage, Birth preparedness and complication readiness (BPCR) interventions are effective in reducing maternal and neonatal mortality in low resources settings [3].

Cameroon is one of the many countries in Africa that has the BPCR plan included in its antenatal consultation (ANC). The BPCR plan has nine elements that are to help the pregnant woman and the family to urgently intervene in case of complications and delivery. The importance of the BPCR intervention in the reduction of maternal mortality has been considered by the Ministry of Public Health (MPH) so that health professions are being trained in the provision of FANC. In the North West Region (NWR), the proportion of women who attend ANC provided by trained health personnel in the North West Region stands at 97.6%. Education on danger signs in pregnancy and childbirth is a part of the BPCR strategy. However, information on possible complications in pregnancy was received by 77.1% of women who had received information on danger signs during pregnancy according to the 2011 Cameroon Demographic Health Survey [4]. Even though birth preparedness and complication readiness is an integral part of FANC to reduce maternal mortality in Cameroon, little or no information is available on the factors associated with birth preparedness and complication readiness among pregnant women attending ANC in Cameroon, and particularly in Bamenda. This study, therefore, aimed to assess the factors associated with birth preparedness and complication readiness among pregnant women attending government health facilities in the Bamenda Health District.

## Methods

**Study design:** this was an analytic health facility-based cross-sectional study.

**Duration and period of study:** the duration of this study took eleven months (February - December 2016) while the period of the study being for data collection was a month (30^th^ October to 30^th^ November 2016).

**Study population:** the target population was pregnant women who were at 32 weeks of pregnancy and above and attending ANC at chosen government health facilities in the Bamenda Health District.

**Criteria of inclusion:** willing and able to give informed consent. Women who are ≥ 32 weeks of pregnancy and attending ANC at the chosen health facilities.

**Criteria of exclusion:** women were excluded from the study if: they have attended only one ANC visit.

**Sampling method:** the method of sampling used in this study was the non-probabilistic convenience method. Sample size to calculate our sample size, we used the formula

$$N=\frac{2P(1-P){\left(Z\alpha +Z\beta \right)}^{2}}{P0-P1^{2}}$$

Where P = (P0 + P1)/2; P0 = proportion of pregnant women who were prepared and knowledgeable on danger signs (0.821); P1= proportion of pregnant women who were unprepared and knowledgeable on danger signs (0.602); α = 0. 05 → Zα = 1.65; β = 0. 1 → Zβ = 1.28; P = (0.821 + 0.602)/2 = 0.7115; N = 74; therefore, total sample size is 148. A total of 345 participants were finally included in this study.

### Variables

**Dependent variable:** the dependent variable was birth preparedness and complication readiness. The women were grouped as “prepared” and “unprepared”. Women were considered “prepared” on these seven aspects of BPCR (identify health facility, saved funds for birth/complications, means of transportation in birth/emergency, identify blood donors, packed necessary materials for birth, identified decision-maker and birth companion) while those who had done less than these seven aspects were “unprepared”.

**Independent variables:** the independent variables to be associated with the dependent variable are: socio-demographic factors (age, level of education, marital status, religion, residence, income); reproductive health factors (parity, gestational age at first ANC, and number of ANC visits)

**Data collection tools/technique:** the tool for data collection was a structured questionnaire to be filled by the investigator. Two state registered nurses were trained and helped in the data collection. The technique of the data collection was through a face to face interview with the participant to elicit responses if eligibility for the study within the period of data collection. Although the original questionnaires were in the English language, pidgin English had to be used at times for those who could not understand English.

**Pre-test and validation of data collection tools:** the researcher used this procedure to assure the feasibility and validity of the data collecting tool. It is a trial process to determine if the data collecting tool can permit to collect reliable data. This tool is elaborated by the student herself and submitted to the research director for correction and validation.

**Data processing and analysis:** data collected were coded and entered into Microsoft Excel. It was then transferred and analysed using Statistics is a software package (SPSS) version 20 software. To be able to provide an adequate response to the specific objectives, a descriptive analysis with Pearson´s chi-square was done to get a statistical significant association between BPCR and socio-demographic and reproductive health characteristics. The statistically significant variables were included in logistic regression. Both bivariate and multivariate (simultaneously with all significant independent variables) logistic regression at 95% confidence level and p < 0.05 were done to identify factors that favour birth preparedness and complication readiness.

**Ethical consideration:** ethical clearance was obtained from the ethical review board of the School of Health Sciences, Catholic University of Central Africa. An administrative authorization from the Regional delegate of Public Health, North West Region was obtained. A notice of information to the participants assuring them of the academic purpose of this study and the confidentiality of their respond was effected.

## Results

A total of 345 pregnant women were included in the interview. They comprised of pregnant women with gestational age ≥ 32weeks. The modal age range was 25 - 34 years with the least age being 15 years and the most being 44 years. The respondents comprised of 2.3% who had no formal education, 29.6% who had gone through primary education, 47% with secondary education, and 21.1% who had received a university education. More than half of the respondents were married (65.5%), 21.5% were single, and 13% were cohabitating. Most of the women interviewed were self-employed (42.3%), then students (18.6%), 11.3% private employed, 11% housewives, 10.7% were government employed, and 6.1% had other occupations or did nothing. The socio-economic status of the women was partitioned into low, moderate, and high according to their monthly income. The majority of the respondents were Christians (96.5%) and then Muslims (3.5%). Sixty-six point one (66.1%) were residents in the urban areas and 33.9% in the rural areas (Table 1). Concerning the reproductive health characteristics 34.5% had not given birth yet, 57.1% have given birth at least three times, and 8.4% have had four and more deliveries. Several of these women begin their first ANC visit during the second trimester (67%), 31% started before 16 weeks, and 2% during the third trimester. The mean gestational age at first ANC was approximately 19 weeks. Seventy-one-point one percent (71.1%) had attended less than four ANC and 28.9% more than four ANC visits.

**Table 1:** characteristics of birth preparedness and complication readiness

Characteristics	Birth preparedness and complication readiness N = 345		Bivariate OR (95% CI)	P-value	Multivariate OR (95%CI)
	Unprepared (N = 280 n (%)	Prepared (N = 65 n (%)			
**Age**					
15-24	65(23.2)	17(26.1)	1c		1c
25-34	173(61.8)	38(58.5)	7.43(0.95, 58.40)	0.057	5.10(0.58, 45.12)
	0.143				
35-44	42(15.0)	10(15.4)	6.57(0.52, 83.76)	0.147	7.42(0.51,108.41)
	0.143				
**Level of education**					
Primary and below	101(36.1)	9(13.8)	1c		1c
Secondary	132(47.1)	30(46.1)	2.55(1.16, 5.61)	0.02	2.1(0.92, 4.8)
	0.079				
University	47(16.8)	26(40.0)	6.21(2.70, 14.28)	0.001	2.5(0.95, 6.73)
	0.064				
**Occupation**					
No employment	101(36.1)	22(33.8)	1c		1c
Gov't employee	21(7.5)	16(24.6)	3.50(1.58, 7.76)	0.002	0.75(0.18, 3.15)
	0.699				
Private/self-employee	158(56.4)	27(41.5)	0.79(0.4, 1.45)	0.44	0.50(0.24, 1.02)
	0.056				
**Monthly income**					
0 - 49 999	213(76.1)	28(43.1)	1c		1c
50 000 - 99 999	64(22.8)	33(50.8)	3.29(1.69, 6.40)	0.001	2.94(1.39, 6.25)
	0.005				
100 000 - 300 000	3(1.1)	4(6.1)	5.95(2.86, 12.38)	0.001	3.0(0.81, 11.68)
	0.1				
**Residence**					
Rural	100(35.7)	17(26.2)	1c		1c
Urban	180(64.3)	48(73.8)	1.57(0.86, 2.87)	0.145	1.63(0.82, 3.22)
	0.162				
**Number of ANC visits**					
2. - 3	209(74.6)	36(55.4)	1c		1c
4. - 9	71(25.4)	29(44.6)	2.37(1.35, 4.14)	0.002	2.16(1.18, 3.90)
	0.013				
**Knowledge on danger signs**					
0 - 2	198(70.7)	36(55.4)	1c		1c
3 - 5	82(29.3)	29(44.6)	1.95(1.12, 3.38)*	0.018	1.39(0.75, 2.58)
	0.294				

In the bivariate logistic regression analysis of the socio-demographic and reproductive health characteristics of the respondents, level of education, occupation, monthly income, number of ANC visits, and knowledge of danger signs in pregnancy were significantly associated with birth preparedness and complication readiness. Pregnant women who were government employed were 3.5 times more likely to prepare for birth and complications than those who did not have employment (OR = 3.50, 95% CI: 1.58, 7.76, p = 0.002). However, those who were private/self-employed were less likely to prepare for birth/complications. Women with University education were 6.2 times (OR = 6.21, 95% CI: 2.70, 14.28, p = 0.001) more likely to prepare for birth and its complication and those with secondary education 2.5 times (OR = 2.55, 95% CI: 1.16, 5.61, p = 0.020) than women who had primary education and below. This study revealed that women with high monthly income of at least 100.000 francs were 5.95 times (OR = 5.95, 95% CI: 2.86, 12.38, p = 0.001) more likely to prepare for birth and its complication and those with moderate monthly income 50 000 - 99 999 francs 3.29 times (OR = 3.29, 95% CI: 1.69, p = 0.001) than women with monthly income below 50 000 francs. Respondents who could spontaneously give at least three danger signs in pregnancy were considered knowledgeable. Those who were considered knowledgeable were two times more likely to prepare for birth and complications than those who knew less than three danger signs (OR = 1.99, 95% CI: 1.12, 3.38, p = 0.018). Concerning the number of ANC visits, women who had attended at least four antenatal visits were 2.37 times more likely to prepare for birth and its complication than those who had less than four visits (OR = 2.37, 95% CI: 1.35, 4.14, p = 0.002).

In the multivariate logistic regression was done using the socio-demographic and reproductive health characteristics, only monthly income (p = 0.013) and the number of ANC visits (p = 0.013) were significantly associated with birth preparedness and complication readiness. Women with high monthly income were approximately three times more likely to prepare for birth and complications (OR = 2.942, 95% CI: 1.39, 6.25, p = 0.005). Those with at least four ANC visits attendance were two times to prepare for birth and complications (OR = 2.16, 95% CI = 1.18, 3.90, p = 0.013).

## Discussion

Findings from this study have shown the association of certain socio-demographic and reproductive health characteristics with birth preparedness and complication readiness. In this study the factors associated with BPCR on bivariate analysis were; level of education, occupation, income, number of ANC visits, and knowledge of danger signs in pregnancy. The level of education was statistically associated with birth preparedness on bivariate analysis as revealed in this study. Those in the University were six times more likely to prepare for birth or complications in pregnancy (OR = 6.21, 95% CI: 2.70, 14.28, p = 0.001) compared to those with primary level and below. Similarities to this finding were seen in studies carried out in Kenya and Nigeria [5,6]. In Uganda and Ethiopia, studies [7,8] found education as a predictor of birth preparedness and complication readiness after a multivariate analysis which is inconsistent with the findings of this study. The ability of educated women to make decisions on issues concerning their health can explain this phenomenon. More educated mothers tend to have better awareness of warning signs of obstetric complications. It also might be related to the fact that educated women have a better power to make their own decision in matters related to their health and the expected expenses. The results indicated that the level of education influenced the ability and the quality of decisions on birth preparedness and complication readiness. Women with a higher level of education are more able to make better decisions on birth preparedness. Therefore, the education of women should be encouraged in the community which shall be beneficial to their health. The occupation status was also associated with birth preparedness and complication readiness on bivariate analysis. The occupation is the most probable steady source of income for an individual. In this study, women who were employed by the government were 3.5times more likely to prepare for birth and complications than those with no employment (students, housewives, farmers). This finding is quite similar to a study in Ethiopia [9]. Other studies also confirm that women who are employed are more likely to prepare for birth and complications [10,11]. Given that one´s occupation may determine the income, government employees in Cameroon are considered to be well paid than those of the private sector. This may explain why they are more prepared. However, the controversy in this study lies in the fact that those who are self-employed are less likely to be prepared compared to those with no employment.

Monthly income was associated with birth preparedness and complication readiness. This is consistent with studies in Kenya, Nigeria, India, and Uganda [12-15] respectively. Income is an important element in the preparation of birth/complications. It is necessary to take care of the cost of delivery, transportation, and other requirements related to pregnancy. An increase in the average income of an individual has a positive influence on the likelihood of preparing for birth and its complications. This relates to the three delays model where the first delay (decision to seek care) is related to socio-economic and cultural factors. This could be because the economic status of the woman gives her the ability to make wise decisions and payments on her own than their counterparts. Given that some of the elements of the BPCR plan require finances to be prepared, the pregnant women with available financial resources will more likely prepare for birth and complications as it will not be seen as a difficult task. In the theory of planned behaviour (TPB), Fishbein and Ajzen [16] explained that an individual will easily engage in healthy behaviour when it is easy to perform that particular behaviour.

The number of ANC visits is also statistically significant to birth preparedness (OR = 2.16, 95% CI: 1.18, 3.90, p = 0.013) following the multivariate OR. This is similar to studies in Tanzania [10,11] where women who attended at least four ANC visits were approximately two times more prepared than women who attended less. The benefits of ANC to the health of the mother and the baby should have been perceived by the women who frequent ANC visits more. This action will expose the women more to information provided by the health professionals. To explain this likelihood, it is thought that women who attend more ANC visits are repeatedly exposed to information provided by the health professionals. This information received as the importance of BPCR is capable to make them adopt behaviours of preparation for pregnancy and possible complications in pregnancy. This information also acts as external stimuli to trigger the pregnant women into the action of BPCR. This can be explained by the construct of cues to action elaborated in the Health Belief Model (HBM) by Rosenstock *et al*. [17] and behavioural intention of the theory of planned behaviour [18] where information provided on BPCR.

One of the basic assumptions stipulated by the concept of BPCR is the fact that the knowledge of danger signs will lead to a greater preparation to minimize the effects of complications in pregnancy and childbirth. This is so by reducing the first two delays common in untimely care. In this study, women who knew obstetrics danger signs were more likely to be prepared for birth and its complication compared to those who did not know. The finding of this study showed that knowledge of danger signs is associated with BPCR (OR = 1.95, 95% CI 1.12, 3.38; p = 0.018). This is similar to studies in Nigeria, Ethiopia, India, and Uganda [13,19-21]. In this study, those knowledgeable of danger signs were 1.9.6 times more likely to be prepared than those without. The reason for this might be mothers with knowledge of obstetric complications may fear something may happen and need advice and support from health personnel. According to Glanz *et al*. [17] in their description of the HBM when an individual thinks there is a possibility to acquire a health problem and perceives that the severity of the health problem can alter his normal social life, then there is a high probability to adopt beneficial health behaviours.

**Study limitation:** the study was health facility-based which might not indicate the true proportion of BPCR practice in this health district. Also, the fact that information related to the level of birth preparedness and complication readiness was obtained through interviews could be limiting as what people say may not necessarily be what they practice. Despite these limitations, the findings from this study will contribute to the understanding of the factors associated with BPCR practice in the study area

## Conclusion

Complications leading to maternal death are unpredictable; occurring without warning at any time during pregnancy and childbirth. These deaths can be minimized if adequate preparations had been made to overcome the delays to seek care. The major reason for which the strategy of birth preparedness and complication readiness was involved in antenatal consultation. This study identified factors that could have a positive influence on birth preparedness and complication readiness in the Bamenda Health District. Socio-demographic and reproductive health characteristics of monthly income and number of antenatal visits were associated with birth preparedness and complication readiness. Effective education on the importance of birth preparedness and complication readiness needs to be exercised in the various health facilities so that the barriers of implementation can be overcome. The media and other communications mediums in the community will be appropriate avenues to propagate this tool that can be effective in the reduction of maternal deaths.

### What is known about this topic

- Knowledge on danger signs in pregnancy among pregnant women;
- Utilization of antenatal care services and quality of care provided at antenatal care services;
- Perceptions of antenatal care services by pregnant women attending government health centres in the Buea Health District, Cameroon.

### What this study adds

- The findings from this study revealed that the strategy of BPCR has not been fully immersed in the health services so that its impact can be optimised.
